# Pro-myogenic small molecules revealed by a chemical screen on primary muscle stem cells

**DOI:** 10.1186/s13395-020-00248-z

**Published:** 2020-10-09

**Authors:** Sean M. Buchanan, Feodor D. Price, Alessandra Castiglioni, Amanda Wagner Gee, Joel Schneider, Mark N. Matyas, Monica Hayhurst, Mohammadsharif Tabebordbar, Amy J. Wagers, Lee L. Rubin

**Affiliations:** 1 Harvard University Department of Stem Cell and Regenerative Biology, 7 Divinity Ave, Cambridge, MA 02138 USA; 2 Cancer Immunology Department, Genentech, 1 DNA Way, South San Francisco, CA 94080 USA

**Keywords:** Satellite cells, Screening, RET, GDNF, Lestaurtinib, CEP-701

## Abstract

Satellite cells are the canonical muscle stem cells that regenerate damaged skeletal muscle. Loss of function of these cells has been linked to reduced muscle repair capacity and compromised muscle health in acute muscle injury and congenital neuromuscular diseases. To identify new pathways that can prevent loss of skeletal muscle function or enhance regenerative potential, we established an imaging-based screen capable of identifying small molecules that promote the expansion of freshly isolated satellite cells. We found several classes of receptor tyrosine kinase (RTK) inhibitors that increased freshly isolated satellite cell numbers in vitro. Further exploration of one of these compounds, the RTK inhibitor CEP-701 (also known as lestaurtinib), revealed potent activity on mouse satellite cells both in vitro and in vivo. This expansion potential was not seen upon exposure of proliferating committed myoblasts or non-myogenic fibroblasts to CEP-701. When delivered subcutaneously to acutely injured animals, CEP-701 increased both the total number of satellite cells and the rate of muscle repair, as revealed by an increased cross-sectional area of regenerating fibers. Moreover, freshly isolated satellite cells expanded ex vivo in the presence of CEP-701 displayed enhanced muscle engraftment potential upon in vivo transplantation. We provide compelling evidence that certain RTKs, and in particular RET, regulate satellite cell expansion during muscle regeneration. This study demonstrates the power of small molecule screens of even rare adult stem cell populations for identifying stem cell-targeting compounds with therapeutic potential.

## Background

Satellite cells are the adult stem cells of skeletal muscle and support postnatal muscle growth and repair. In adult muscle, satellite cells are, for the most part, mitotically quiescent and reside between the basal lamina and sarcolemma of the muscle fiber [1–3]. Satellite cells can both differentiate and self-renew, and these characteristics allow adult skeletal muscle to regenerate repeatedly in the face of muscle damage [4–6]. Following acute injury, satellite cells exit quiescence and give rise to a transit-amplifying population of myogenic progenitors termed myoblasts [7, 8]. The capacity of the skeletal muscle to grow and regenerate is also required during postnatal growth [9, 10], and the ability to maintain and regenerate muscle is impaired during the aging process and several neuromuscular disorders [11–14].

One frequently discussed therapeutic approach to treating muscle damage or deterioration is to graft large numbers of satellite cells into the affected tissue [4]. However, the limited ability to produce the necessary numbers of engraftable cells, coupled with the challenges of intramuscular delivery, presents practical hurdles for this strategy. The second, potentially complementary, idea is to find agents that stimulate the intrinsic regenerative capacity of endogenous satellite cells, for instance, by targeting growth factors and/or hormones that regulate this process or at least allow for the ex vivo expansion of these cells [15–17].

In this study, we developed and conducted a chemical screen using highly purified satellite cells freshly isolated from adult mouse skeletal muscle to discover agents capable of increasing satellite cell numbers. We found several small molecule inhibitors of receptor tyrosine kinases (RTKs) that promote satellite cell expansion. Further analysis of one of these compounds, CEP-701 (also known as lestaurtinib), revealed a potent, dose-dependent ability to increase the numbers of freshly isolated satellite cells cultured ex vivo. Of the cell types tested, this activity was relatively specific to satellite cells as neither myoblasts nor interstitial fibroblasts were expanded by CEP-701 treatment, and the proliferation of both THP-1 and Neuro2a was inhibited. Furthermore, adult satellite cells, freshly isolated from skeletal muscle and treated with CEP-701 ex vivo for 5 days, exhibited enhanced muscle engraftment efficiency, as compared to vehicle-treated controls. Consistent with these results, systemic administration of CEP-701 following muscle injury enhanced myofiber regeneration and was accompanied by an in vivo expansion of satellite cell numbers. Based on the putative targets of CEP-701 [18], we identified the proto-oncogene RET as the predominant RTK present on adult satellite cells and showed that the RET ligand GDNF can inhibit satellite cell proliferation in vitro. Together, these results implicate RTK signaling, and more specifically signaling via GDNF/RET, in skeletal muscle regeneration. Furthermore, this work’s identification of RET inhibitors as candidate therapeutics demonstrates the utility of performing direct chemical screening on primary stem cell populations to treat muscle-wasting conditions.

## Methods

### Animal experiments

For this study, we used the following mouse lines bred in our animal facility: chicken b-actin GFP transgenic mice (C57BL/Ka-b-actin-EGFP) [19], *mdx* mice (C57BL/10ScSn-Dmd^*mdx*^/J—Jackson Laboratories, Bar Harbor, ME—#001801) [20], Tg:Pax7^nGFP^ mice [21], FLT3 KO mice (kindly provided by Camilla Forsberg; UC Santa Cruz), and C56BL/6 mice (Charles River Laboratories). All mice were housed under standard conditions and allowed free access to food and water ad libitum. All experiments were performed in accordance with the Harvard University guidelines for animal handling and animal care determined by the Harvard University Animal Care Committee.

### Human skeletal muscle specimens

The human fetal muscle was obtained from 20- to 23-week gestation fetuses. Harvard IRB approval was awarded to conduct the experiments in this study.

### Primary cell isolation

Mouse satellite cells from C57BL/6 mice were isolated from intact limb muscle of 2–4-month-old animals and prepared for fluorescence-activated cell sorting (FACS) based on the cell surface marker profile CD45^−^/Ter119^−^/CD11b^−^/Sca1^−^/CXCR4^+^/ITGB1^+^, as described previously [22–24]. Mouse satellite cells from Tg:Pax7^nGFP^ mice were FACS-isolated from the intact limb muscle of 2–4-month-old animals based on their nGFP expression. Experiments with freshly isolated satellite cells were carried out within the first 1–6 days post-isolation. CD45^−^/CD11b^−^/GlyA^−^/CD31^−^/CD34^−^/CD56^int^/ITGA7^hi^ human satellite cells were isolated from human fetal muscle as described previously [25]. Mouse fibroadipogenic precursor cells (FAPs) were isolated as CD45^−^/Ter119^−^/CD11b^−^/Sca1^+^/CXCR4^−^/ITGB1^−^ cells as described previously [26]. Blood lineage cells were isolated as the pooled population of Sca1^−^/CXCR4^−^/ITGB1^−^ cells that express CD45, Ter119, or CD11b. Committed myoblast lines used in this study refer to purified satellite cells that were maintained under proliferative conditions in vitro for > 2 weeks. Primary interstitial fibroblasts were isolated as a byproduct during muscle digestion from Tg:Pax7^nGFP^ and were FACS-sorted to exclude nGFP. FACS and cell analysis was performed at the Harvard Stem Cell Institute Flow Cytometry Core Facility on a BD FACSAria II (BD Biosciences, San Jose, CA).

### In vitro culture and chemical screening

**Fig. 1:**
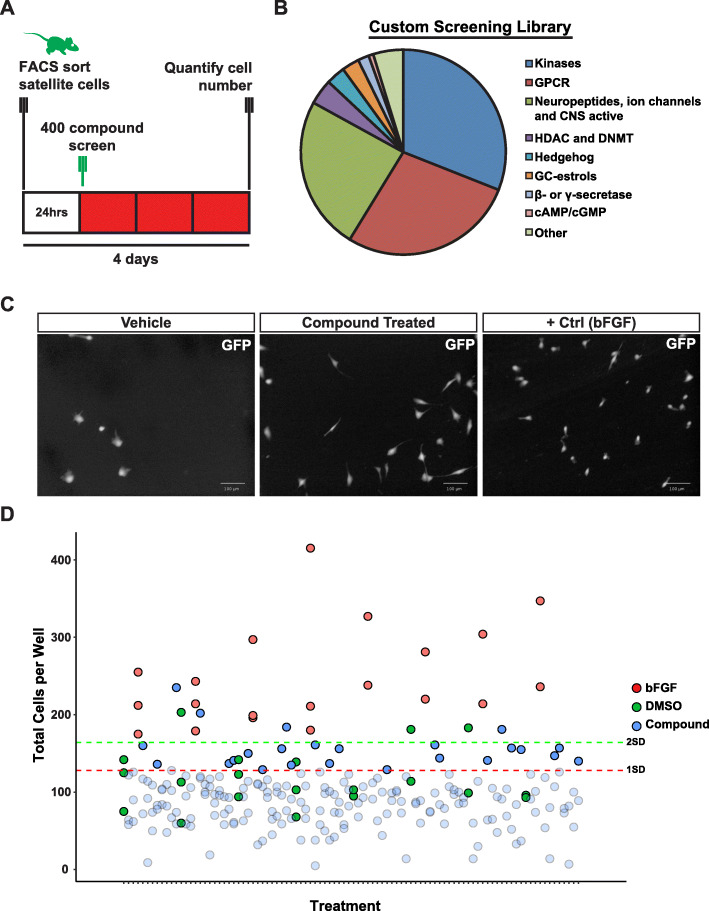
Identification of small molecules that promote satellite cell proliferation. **a** Chemical screen experimental schematic outlining FACS isolation and compound library treatment of satellite cells. **b** Screening compound library subdivided into broad target classes. **c** Representative immunofluorescent images of FACS-sorted satellite cells from C57BL/6-β-actin-EGFP mice on 96-well plates cultured for 4 days and treated with vehicle, compound, or bFGF. Proliferation was assessed via high content imaging using GFP as a cell marker. Scale bars denote 100 μm. **d** Change in total satellite cell number after culture in the presence of DMSO (green), bFGF (red), or screening compounds (blue). Dashed lines indicated 1 standard deviation (red) or 2 standard deviations (green) above the global mean calculated across all plates. Compounds that fall below 1 standard deviation from the vehicle mean are depicted in shaded blue

**Fig. 2:**
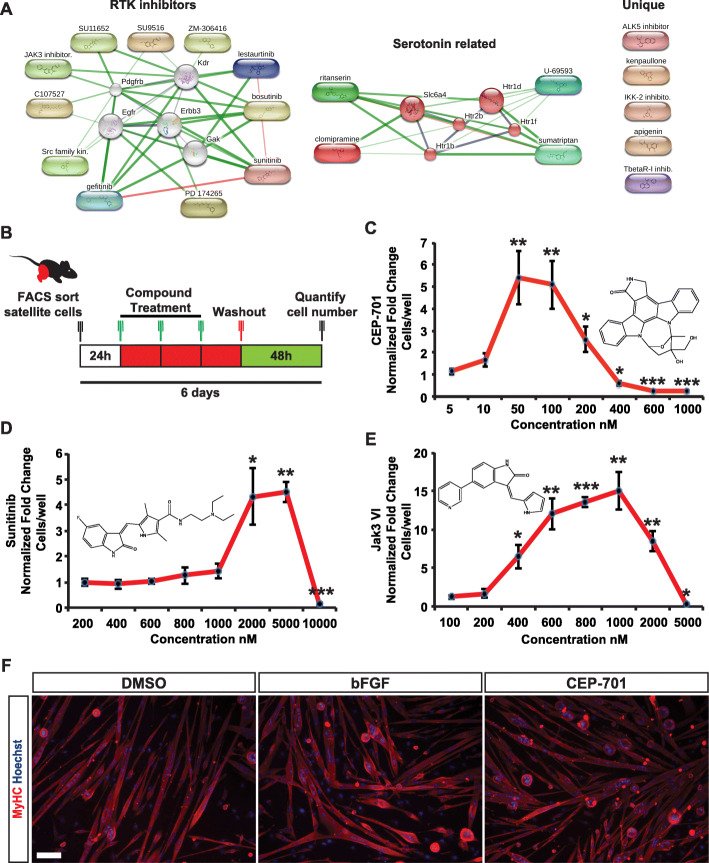
Validation and dose-response of chemical screen hits. **a** STITCH interaction networks generated from the primary screen hit compounds. **b** Experimental schematic detailing the compound treatment and culture duration for dose-response analysis. **c**–**e** Dose-response curves for hits present in the primary compound library. Error bars indicate SD from at least three independent experiments. Statistical significance was evaluated by a one-way ANOVA followed by post hoc Welch’s *t* test (*p < 0.05, **p < 0.01, ***p < 0.001). ANOVA *p* values for the following compounds: CEP-701 = 1.82E−11, Sunitinib = 7.38E−11, and Jak3 VI = 2.43E−12. **f** FACS-sorted satellite cells, expanded in the presence of either vehicle, CEP-701 (50 nM), or bFGF (5 ng/mL) then differentiated for 5 days, retain the capacity to fuse and form multinucleated myotubes. Myosin heavy chain (MyHC) is stained in red while the nuclei are counterstained with Hoechst (blue). Scale bar represents 100 μm

Freshly FACS-isolated satellite cells were plated in 96-well plates pre-coated with laminin in a growth medium containing Ham’s F10 nutrient mixture (Life Technologies or Wisent Bioproducts), 10% heat-inactivated horse serum (Life Technologies), 1× penicillin/streptomycin (Life Technologies), and 1× l-glutamine (Life Technologies). Notably, our growth media do not contain bFGF. For screening, cells were plated at 50 cells/well. For follow-up assays, cell density was 300 cells/well unless noted otherwise. The day cells were plated was considered day 0. Compounds were added on day 1. For screening, cells were incubated without medium change until day 4, and plates were fixed and imaged (Fig. 1a). For follow-up assays, media were changed and fresh compounds added on days 2 and 3, then compounds were washed out on day 4. The plates were incubated in a growth medium, once again in the absence of bFGF, and then fixed for imaging and analysis on day 6 (Fig. 2b). All compounds were resuspended in dimethylsulfoxide (DMSO, Sigma-Aldrich); 0.1% DMSO was added as a negative control in all assays; 5 ng/mL basic fibroblast growth factor (bFGF, Life Technologies) was added as a positive control in all assays. For differentiation assays in Fig. 2f, cells were cultured for an additional 6–8 days in low-serum differentiation media.

Proliferating committed myoblasts were seeded at 2000 cells/well in 96-well plates with or without 5 ng/mL bFGF in Ham’s F10, 20% FBS, GlutaMAX, and non-essential amino acids. Compounds were added, and media were changed to match our assay used to screen freshly isolated satellite cells (Fig. 2b). Differentiation assays were carried out by seeding 10,000 myoblasts per well and culturing in DMEM, 5% horse serum, GlutaMAX, and non-essential amino acids for 2 days. Lestaurtinib (CEP-701) was purchased from LC Laboratories (Cat# L-6307) while sunitinib (Cat# S7781) and Jak3 inhibitor VI (Cat# 420126) were purchased from Selleck Chemicals and Sigma-Aldrich, respectively. Primary interstitial fibroblasts were seeded at 200 cells/well in 96-well plates and proliferated with or without 5 ng/mL bFGF in DMEM, 10% FBS, and GlutaMAX to match the assay used to screen freshly isolated satellite cells (Fig. 2b).

Neuro-2A (ATCC # CCL-131) were seeded at 1000 cells/well in 96-well plates in DMEM and 10% FBS. After 24 h, compounds were added once and cells were cultured for an additional 7 days before fixation and counting by Hoechst stain.

THP-1 (ATCC # TIB-202) were seeded at 2500 cells/well in 96-well plates in RPMI, 10% FBS, GlutaMAX, and β-mercaptoethanol. After 24 h, compounds were added once and cells were cultured for an additional 7 days before measuring proliferation by MTT assay (Sigma-Aldrich/Roche # 11465007001) according to the manufacturer’s instructions.

### Engraftment of freshly isolated satellite cells following ex vivo expansion with CEP-701

On day 0, freshly isolated satellite cells were sorted from the Ka-b-actin-EGFP mice. On days 1, 2, and 3, cells were treated with 50 nM CEP-701 or DMSO. On day 5, cells were harvested and counted; 30,000 cells were then injected into mdx TA muscle (injured with cardiotoxin 24 h before). After 21 days, the muscle was collected, fixed in 4% PFA for 1 h, put in 30% sucrose for 6 h, and then frozen. The muscles were then cryo-sectioned: for each muscle, we collected 12 sections separated by 240 μm and counted the number of GFP^+^ fibers per section.

### Muscle injury and CEP-701 delivery

Muscle injury was performed as described previously [5]. Briefly, 50μL injections of a 10μM solution of cardiotoxin (CTX) were injected into the tibialis anterior (TA) muscle of isoflurane-anesthetized C57Bl/6 male mice. CEP-701 dissolved in 40% PEG 1000/10% povidone was administered subcutaneously at a dose of 10 mg/kg based on the pre-clinical work published by Zarrinkar et al. twice daily for a period of 4 days. In specific experiments, satellite cells were quantified by flow cytometry at day 2. In others, we collected the muscles at day 4 and stained for eMHC.

### Myofiber culture

Myofiber culture and isolation were performed as described previously [27]. Enumeration of ≥ 50 satellite cells was conducted across a minimum of 15 fibers per replicate at each time point (0, 24, 48, and 72 h). These experiments were conducted in at least biological triplicate from each condition at each time point using Tg:Pax7^nGFP^ male mice.

### Immunofluorescence and image analysis

Cells, ex vivo muscle fibers, and tissue sections were fixed with 4% paraformaldehyde solution (PFA, Electron Microscopy Science) for 10–15 min at room temperature and washed with Dulbecco’s phosphate-buffered saline (DPBS, Life Technologies) 3 times for 5 min. Cells were identified and counted either by innately expressed GFP (where appropriate) or Hoechst stain. Where indicated, antibody stains were performed as follows. Samples were permeabilized in 0.2% Triton-X (Sigma-Aldrich) at room temperature for 20 min, then blocked in 5% goat serum, 5% horse serum (Life Technologies), and M.O.M. blocking agent (where appropriate; Vector Laboratories) for 1 h at room temperature. Samples were incubated with primary antibodies overnight at 4 °C in block. Antibody concentrations were 1:2 Pax7 hybridoma supernatant (Developmental Studies Hybridoma Bank at the University of Iowa), 1:100 MyoD (clone G-1, Santa Cruz Biotechnology sc-377460), 1:100 Ki67 (clone SolA15, Thermo Fisher 14-5698-82), 1:10 embryonic myosin hybridoma supernatant (DSHB F1.652), 1:10 myosin heavy chain hybridoma supernatant (DSHB MF20), 1:1000 laminin (Sigma L9393), 1:400 RET (Neuromics GT15002) or 1:100 (Abcam ab134100), 1:100 phospho-RET (Santa Cruz Biotechnology sc-20252-R), and 1:100 GFRα1 (Abcam ab8026). Phalloidin-488 was used at 1:200 in PBS (Thermo Fisher A12379). Cells were washed 3 times for 5 min with DPBS and incubated in secondary antibody for 1 h at room temperature using 1:2000 Alexa Fluor 488, 546, or 647 (Life Technologies) then washed. The nuclei were stained with 1:5000 Hoechst 33342, trihydrochloride, and trihydrate (Life Technologies) for 5 min at room temperature.

Images were acquired using the Opera automated confocal imager or Operetta automated epifluorescent imager (Perkin Elmer) as described previously [28, 29], the Molecular Devices ImageXpress, the Nikon Ti automated imager with LED illumination, or the Zeiss LSM880 confocal microscope. Images were analyzed with Perkin Elmer Columbus Image Data Storage and Analysis System v2.3.3 or NIS Elements BR3.0.

### Bioinformatics

The following microarray datasets were obtained from GEO. Transcriptional profiles were obtained from freshly isolated satellite cells with respect to age (3 months and 18 months) GSE47401. Microarray specifics and data processing were previously published [30]. Proliferating myoblasts and myotubes differentiated for 5 days GSE24811. Microarray specifics and data processing were previously published [31]. The RMA expression values are log2-transformed. Heatmaps were generated by Gene-Pattern www.broadinstitute.org/cancer/software/genepattern.

## Results

### A small molecule phenotypic screen identifies compounds that expand freshly isolated satellite cells

We conducted a chemical screen to identify compounds that can expand freshly isolated satellite cells in vitro. Primary satellite cells were isolated from 3-month-old adult C57BL/6-β-actin-EGFP mouse muscle by enzymatic digestion followed by fluorescence-activated cell sorting (FACS) to select cells exhibiting the surface marker phenotype CD45^−^/TER119^−^/CD11b^−^/SCA1^−^/CXCR4^+^/ITGB1^+^, as described previously [22]. Cells isolated based on this CXCR4^+^/ITGB1^+^ immunophenotype exhibit equivalent purity, as determined by Pax7 expression and myogenic potential, as other satellite cell isolation schemes, including sorting for CD34^+^/ITGA7^+^ or for VCAM^+^ cells [24]. FACS-isolated satellite cells were plated in 96-well plates for 24 h before the beginning of compound addition. At this stage, freshly isolated satellite cells have begun to activate, and nearly all cells are positive for Pax7 and MyoD (Fig. S1A-B). In contrast to committed myoblasts, these cells have much lower MyoD expression (Fig. S1B), have not yet begun robust proliferation (Fig. S1C-D), and are smaller in area (Fig. S1E-F), size being an indicator of activation state [32]. Twenty-four hours post-isolation, they were treated in vitro for 3 days with a library of ~ 400 biologically active small molecules composed primarily of kinase inhibitors and GPCR agonists and antagonists (Fig. 1a, b). Hit compounds were defined based on an increase in cell numbers of 1 standard deviation above the vehicle control (Supplementary Table 1). From this screen, we identified multiple compounds that expand freshly isolated satellite cells with activity comparable to that of bFGF, the standard mitogen used to proliferate both myoblasts and satellite cells (Fig. 1c, d) [33, 34].

We next conducted search tool for interactions of chemicals (STITCH) to cluster primary hit compounds based on interactions from metabolic pathways, crystal structures, binding experiments, and drug-target relationships [35]. Using STITCH, we identified two clusters representing signaling pathways that are perturbed by our hit compounds, namely receptor tyrosine kinase (RTK) signaling and serotonin signaling (Fig. 2a). To validate our hit compounds and identify the optimal concentrations at which they act on satellite cells in vitro, we conducted 8-point dose-response curves with freshly isolated satellite cells from wild-type C57BL/6 mice (Fig. 2b–e). The receptor tyrosine kinase (RTK) inhibitor CEP-701 (lestaurtinib) was the most potent of our hit compounds, with a peak effective concentration of 50 nM and an EC50 of ~ 20 nM. Sunitinib, another RTK inhibitor, was active in the low-micromolar range with an EC50 of ~ 1.3 μM and a peak effective concentration of 5 μM. Finally, the compound that produced the greatest cell expansion, giving rise to a 15-fold increase in the number of satellite cells per well relative to the vehicle control, was Jak3 inhibitor VI, at a peak effective concentration of 1 μM and an EC50 of ~ 450 nM. Among these compounds, we chose lestaurtinib (CEP-701) as a compound to be tested further, based on its nanomolar potency, several detailed in vivo studies describing its pharmacodynamic and pharmacokinetic effects ([18, 36–38]) and its prior use in clinical trials [39–41].

We next confirmed that freshly isolated satellite cells when amplified in the presence of CEP-701 retained the ability to form multinucleated myotubes in vitro following differentiation (Fig. 2f). We also investigated the effects of CEP-701 on freshly isolated satellite cells expanded from human fetal tissue [25] (Fig. S2A). In some donor samples, CEP-701 promoted the expansion of human satellite cells at concentrations as low as 1 nM (Fig. S2B-H), a significantly lower concentration compared to doses effective on mouse satellite cells (Fig. 2c).

### Systemic delivery of CEP-701 increases the rate of muscle regeneration

**Fig. 3:**
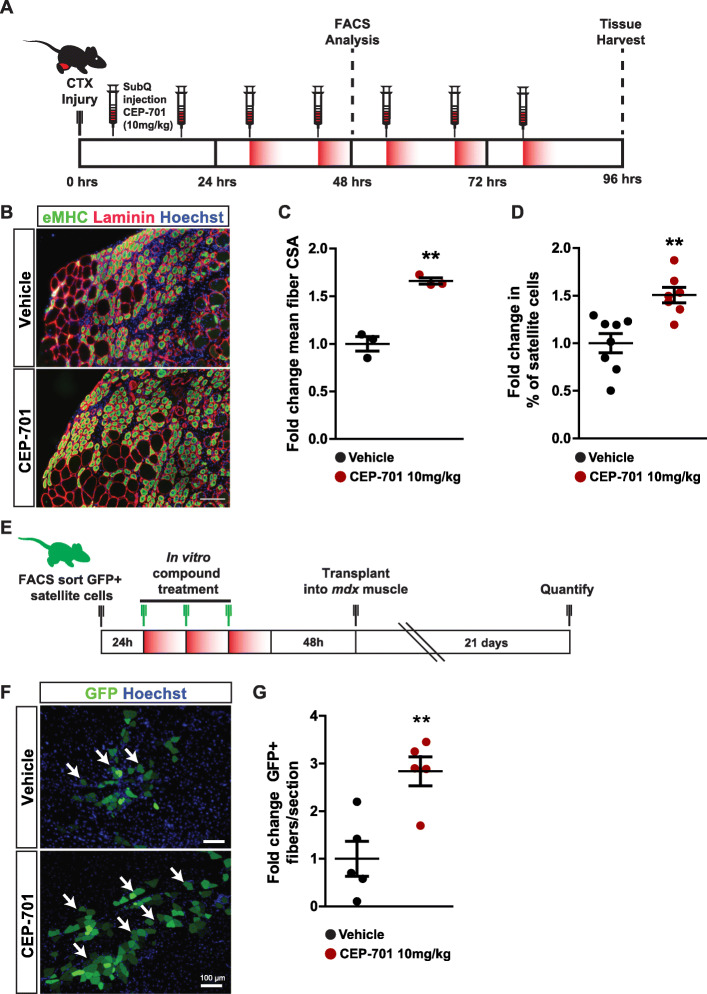
CEP-701 improves muscle regeneration and satellite cell engraftment. **a** Experimental schematic detailing the systemic delivery of CEP-701 to adult (2–4 months old) C57BL/6 mice following CTX-induced TA muscle regeneration. **b** Cardiotoxin (CTX)-induced muscle damage and subsequent regeneration as evidenced by newly formed embryonic myosin-positive (eMHC^+^) fibers. Green, embryonic myosin; red, laminin; blue, Hoechst. Scale bar indicates 100 μm. **c** Quantification of the fold change in the mean cross-sectional area (CSA) of eMHC^+^ regenerating muscle fibers after CEP-701 treatment. The TA muscle was damaged with CTX, and animals were treated subcutaneously, twice a day with vehicle or 10 mg/kg CEP-701. After 4 days, the muscle was isolated, imaged, and analyzed. Error bars indicate SEM from 3 independent experiments. **p < 0.01 by an unpaired two-tailed *t* test assuming unequal variance. **d** Quantification of the fold change in satellite cells in the regenerating muscle following treatment with CEP-701. The TA muscle was damaged with CTX, and animals were treated subcutaneously, twice a day with vehicle or 10 mg/kg CEP-701. CXCR4^+^/ITGβ1^+^ satellite cells were isolated by FACS and quantified as a percentage of the total calcein AM^+^/propidium iodide^-^ live cells. Error bars indicate SEM from 7 independent experiments. **p < 0.01 by an unpaired two-tailed *t* test assuming unequal variance. **e** Experimental schematic outlining ex vivo expansion of satellite cells in the presence of CEP-701 followed by transplantation into regenerating TA muscle of *mdx* mice. **f** Representative images of donor-derived GFP^+^ fibers (green) 3 weeks post-transplantation of CEP-701-treated satellite cells into CTX-injured TA muscle of *mdx* mice. Arrows designate donor-derived myofibers expressing GFP. Scale bar denotes 100 μm. **g** Quantification of engraftment efficiency based on the number of donor-derived myofibers expressing GFP per muscle section. Error bars indicate SEM from 5 independent experiments. **p < 0.01 by an unpaired two-tailed *t* test assuming unequal variance

To investigate whether systemic delivery of CEP-701 affects adult muscle regeneration, we injured the tibialis anterior (TA) muscle of C57BL/6 mice with cardiotoxin (CTX) and then delivered CEP-701 (10 mg/kg) via subcutaneous injection twice daily for 4 days (Fig. 3a). We assessed the speed of regeneration based on the mean cross-sectional area of fibers expressing embryonic myosin heavy chain, a marker for nascent myofibers (Fig. 3b and Fig S3A). Notably, treatment with CEP-701 significantly increased the mean cross-sectional area of regenerating adult myofibers (1.6 ± 0.06-fold) at this early post-injury time point (Fig. 3c and Fig. S3B).

We subsequently investigated whether the administration of CEP-701 affects satellite cells in vivo during muscle regeneration. Consistent with the in vitro expansion effects of CEP-701 on isolated muscle satellite cells, systemic delivery of CEP-701 into mice increased the percentage of satellite cells in regenerating muscle by 50% (Fig. 3d), as measured by flow cytometry 2 days after injury. The percentage of SCA1^+^ fibro/adipogenic progenitors (FAPs, [26]) and of blood lineage cells (the combined percentage of CD11b^+^, TER119^+^, and CD45^+^ cells) did not differ between CEP-treated and vehicle control at the same time point (Fig S3C-D). Taken together, these data suggest that CEP-701 accelerates regeneration in adult skeletal muscle and expands the total pool of regenerative satellite cells available for muscle repair in vivo.

### Ex vivo expansion of satellite cells with CEP-701 promotes engraftment

One of the major hurdles associated with using satellite cells for cell therapy is the loss of their functional capacity to engraft in vivo after they have been isolated and expanded in vitro [3]. To determine if CEP-701 treatment enhances engraftment capacity, we isolated primary satellite cells from C57BL/6-β-actin-EGFP mice [19], expanded them in vitro for 5 days in the presence of 50 nM CEP-701 or vehicle, and transplanted 30,000 expanded donor GFP^+^ cells into regenerating CTX-damaged TA muscle of 6–8-week-old *mdx* mice, a model of Duchenne muscular dystrophy [20] (Fig. 3e). Three weeks after transplantation, the TA muscles from treated mice were dissected and serially sectioned through the mid-belly of the muscle (Fig. 3f). We assessed engraftment based on the number of GFP^+^ myofibers per muscle section (Fig. 3 g) an indication that a donor-derived cell fused with a recipient muscle fiber. The muscles from recipient *mdx* mice transplanted with donor-derived cells treated with CEP-701 contained ~ 3-fold (2.8 ± 0.7) higher numbers of GFP^+^ myofibers compared to recipients that were transplanted with an equal number of vehicle-treated cells (Fig. 3 g), indicating that cells expanded ex vivo in the presence of CEP-701 retain greater engraftment capacity.

### CEP-701 treatment enhances differentiation of committed myoblasts

**Fig. 4:**
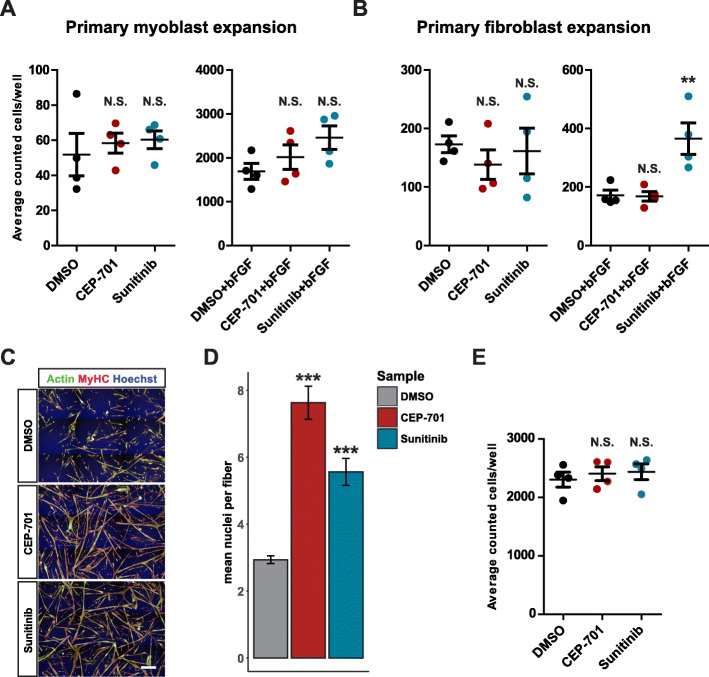
The proliferative effect of CEP-701 is specific to the satellite cell. **a** Culture of primary myoblasts in proliferation conditions with or without bFGF in the presence of 50 nM CEP-701 or 2000 nM sunitinib does not alter expansion. N.S., not significant by 1-way ANOVA. **b** Culture of primary interstitial fibroblasts with or without bFGF in the presence of 50 nM CEP-701 does not alter expansion. Culture without bFGF in the presence of 2000 nM sunitinib does not alter expansion. Culture with bFGF and 2000 nM sunitinib acts synergistically to increase expansion. Both myoblasts and fibroblasts were treated as in Fig. 2a to match the conditions to primary satellite cells. Error bars indicate SEM from 4 independent experiments. **p < 0.001 by 1 way ANOVA followed by unpaired two-tailed *t* test assuming unequal variance with Bonferroni correction for multiple comparisons. **c** Primary myoblasts differentiated for 48 h in the presence of 50 nM CEP-701 or 2000 nM sunitinib demonstrate increased fusion and spindle-like morphology. Green, Phalloidin-488; red, myosin heavy chain; blue, Hoechst. Scale bar represents 200 μm. **d** Quantification of the number of nuclei per cell or fused myotube after myoblast differentiation in the presence or absence of CEP-701 or sunitinib. ***p < 0.001. N.S., not significant by 1-way ANOVA followed by unpaired two-tailed *t* test assuming unequal variance with Bonferroni correction for multiple comparisons. **e** The total number of myoblast nuclei in differentiation conditions is not altered by the presence of 50 nM CEP-701 or 2000 nM sunitinib. Error bars indicate SEM from 4 independent experiments. N.S., not significant by 1-way ANOVA

Upon isolation, satellite cells activate and over time become committed myoblasts that are proliferative and able to further differentiate and fuse to form or repair muscle fibers. A previous publication [42] demonstrated that treating myoblasts with the RTK inhibitor sunitinib promoted the cells’ differentiation capacity in a model of facioscapulohumeral muscular dystrophy (FSHD) [42]. As our data show that sunitinib and CEP-701 can both increase satellite cell numbers in vitro (Fig. 2c, d), we tested whether they might have similar effects on more committed myoblasts. In contrast to our results using freshly isolated satellite cells, neither of these compounds was able to increase the number of committed myoblasts cultured in proliferation media either in the presence or absence of bFGF (Fig. 4a). For CEP-701, this was also true for primary interstitial fibroblasts isolated from the TA muscle (Fig. 4b), while sunitinib failed to expand fibroblasts in base media, but did expand these cells when combined with bFGF (Fig. 4b). As CEP-701 and sunitinib were initially developed as anti-proliferative cancer therapeutics, their effects on satellite cells might be seen as surprising. However, we confirmed the ability of each compound to inhibit the proliferation of acute monocytic leukemia (THP-1) and neuroblastoma (Neuro2a) cells at concentrations equal to those at which they increased satellite cell numbers (Fig S4A-B).

Interestingly, and in agreement with Moyle et al. [42], treatment of myoblasts with CEP-701 or sunitinib in myogenic differentiation conditions gave rise to cells with a more prominent spindle morphology and promoted their differentiation and fusion into multinucleated myotubes (Fig. 4c, d). This effect was not due to a change in the total number of primary myoblasts as neither CEP-701 nor sunitinib expanded these cells during differentiation (Fig. 4e). It is also notable that in vivo, CEP-701 treatment increased the percentage of satellite cells, but did not alter the percentage of FAPs or blood cells in regenerating muscle (Fig. 3d and Fig S3C-D). Taken together, these results suggest that CEP-701 does not promote the expansion of interstitial fibroblasts nor of myoblasts; it can enhance myoblast differentiation and fusion, and of the cell types tested, its pro-expansion effects are restricted to freshly isolated satellite cells.

### CEP-701 targets the RTK RET in satellite cells

To identify possible targets for CEP-701 on muscle satellite cells, we next examined the RTKs predicted to be inhibited by each of our hit compounds (Fig. 2 and Supplementary Table 1) and looked for overlap. Both CEP-701 and sunitinib are predicted to inhibit type III, type IV, and type XIV RTKs including PDGFRα/β, M-CSF1R, FLT3, and RET [18, 43, 44]. CEP-701 was developed as an anti-proliferative FLT3 kinase inhibitor and is effective at inhibiting malignant growth in preclinical models [37, 39, 40]. To test whether FLT3 is involved directly in satellite cell expansion, we conducted proliferation assays using freshly isolated satellite cells from *Flt3* knockout mice. Our results indicate that *Flt3* deletion did not increase basal satellite cell expansion, the opposite of what would be expected if CEP-701 was inhibiting FLT3 in these conditions (Fig. S5A). In addition, *Flt3*^*−/−*^ satellite cells retained the ability to be expanded by culture in the presence of 50 nM CEP-701 (Fig. S5A). Together, these data argue against a role for FLT3 signaling in regulating muscle satellite cell expansion.

**Fig. 5:**
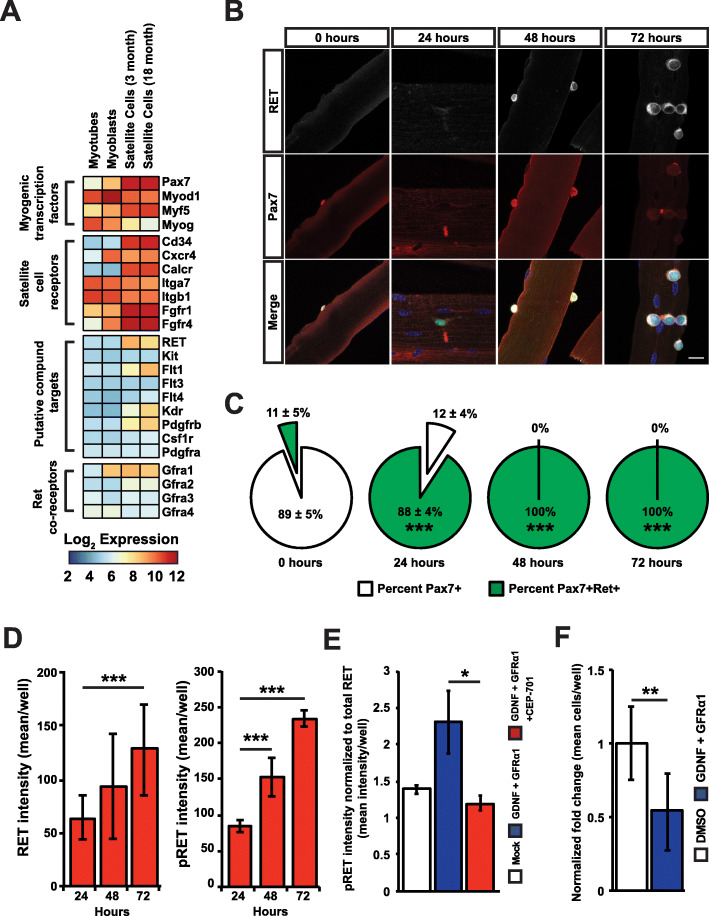
RET is a novel satellite cell receptor inhibited by CEP-701. **a** Fold changes based on log2-transformed RMA values from satellite cells of 3-month, 18-month, cultured myoblast and myotubes used to generate global ratio heat maps. Red indicates genes that increased in expression, and blue indicates a decrease in expression. **b** Representative immunofluorescent images of satellite cells on single muscle fibers isolated from the EDL muscle of adult (2–4 months) Tg:Pax7^nGFP^ mice. Time course conducted at 0, 24, 48, and 72 h of culture in vitro. Cultures were stained with antibodies against RET (white) and Pax7 (red). The nuclei were counterstained with Hoechst (blue). Satellite cells also express GFP under the control of the Pax7 promoter (green). Scale bar indicates 20 μm. **c** Percentage of adult satellite cells (Pax7^+^) expressing RET protein (Pax7^+^/RET^+^) at 0, 24, 48, and 72 h of single EDL muscle fiber culture. Data represent mean ± SD from 3 independent experiments. Statistical significance was evaluated by an unpaired two-tailed *t* test assuming unequal variance relative to (Pax7^+^/RET^+^) 0 h (***p < 0.001). **d** Calculation of RET and phospho-RET intensity on primary CXCR4^+^/ITGβ1^+^ satellite cells isolated by FACS and cultured for 24, 48, and 72 h (**p < 0.01; ***p < 0.001). **e** Phospho-RET intensity normalized to total RET levels in primary CXCR4^+^/ITGβ1^+^ satellite cells isolated by FACS and stimulated with GDNF and GFRα1 either in the presence or absence of 50 nM of CEP-701. *p < 0.05. **f** Normalized fold change in the average number of satellite cells per well following culture in vitro in the presence of 500 ng/mL GDNF and 500 ng/mL GFRα1. *n* = 8. **p < 0.01. Cells were treated as in Fig. 2a to match the conditions to CEP-701-treated cells

To identify possible alternative targets of CEP-701 involved in satellite cell expansion, we assessed published transcriptional profiles of satellite cells, myoblasts, and myotubes [30, 31] for the expression of type III, IV, or XIV RTKs. Of the type III, IV, and XIV RTKs that are putative targets of our hit compounds, expression profiling identified *Flt1*, *Kdr*, *Pdgfrb*, *Ret*, and the *Ret* co-receptor *Gfra1* as expressed in freshly isolated satellite cells (Fig. 5a). To further study RET protein levels and kinetics during satellite cell activation, we isolated single myofibers from 2–4-month-old Tg:Pax7^nGFP^ adult mice and cultured them ex vivo for 72 h [21]. Immediately after isolation (0 h), RET was detectable on 11 ± 5% of satellite cells, and this percentage rose to 88 ± 4% at 24 h and 100% by 48 h (Fig. 5b, c), suggesting that RET expression is absent on most quiescent satellite cells in mature muscle and is upregulated following activation. We next assessed the presence of RET on satellite cells in immature muscle undergoing postnatal myogenesis. Single myofibers were isolated from the muscle of 3-week-old adolescent mice, which are known to have a high basal percentage of activated satellite cells [30]. In these muscles, 55 ± 5% of satellite cells express RET protein at baseline (0 h), approximately 5 times higher than that seen in older animals where postnatal muscle growth has been completed (Fig. S5B). We subsequently confirmed that RET, phosphorylated RET, and the RET co-receptor GFRα1 are all expressed at the protein level and upregulated on primary satellite cells isolated by FACS and cultured in vitro (Fig. 5d, Fig. S5C), whereas we were unable to detect Pdgfrβ at the protein level (not shown). These data indicate that RET protein is more abundant in activated satellite cells and suggest that RET may be involved in the process of satellite cell activation.

To test whether CEP-701 can directly inhibit RET signaling, we stimulated RET phosphorylation in satellite cells cultured for 24 h in vitro with recombinant glial cell line-derived neurotrophic factor (GDNF, 500 ng/mL) along with a soluble form of its co-receptor, GFRα1-Fc (500 ng/mL), and in the presence or absence of 50 nM CEP-701. GDNF exposure led to a 66 ± 18% increase in the levels of activated pRET (Fig. 5e). This increase was blocked by treatment with 50 nM CEP-701 (Fig. 5e). These data suggest that CEP-701 can inhibit RET phosphorylation in satellite cells following stimulation with its physiological ligand GDNF and co-receptor GFRα1. Finally, culturing satellite cells in the presence of GDNF and GFRα1 for 6 days produced an approximately 50% decrease in fold change for satellite cells relative to vehicle control (Fig. 5f). In other words, RET inhibitors stimulate the expansion of satellite cells, while activating RET signaling inhibits their growth.

## Discussion

Satellite cells are the predominant adult stem cell responsible for postnatal muscle growth and regeneration [10, 45, 46]. In diseased muscles, the functional capacity of satellite cells is compromised, leading to inefficient skeletal muscle growth and regeneration capacity [7, 47, 48]. As such, the satellite cell is an excellent target for the development of therapeutics that improve the regenerative capacity of skeletal muscle [4, 49].

In this study, we designed and conducted a small molecule screen that identified compounds capable of expanding satellite cells both in vitro and in vivo. Previous screens on myogenic cell types have identified diverse compounds that affect myogenic induction, regeneration, and differentiation [50–52]. Recent advances in the isolation and characterization of satellite cells have facilitated more physiologically relevant screening assays [53]. Our strategy involved the treatment of freshly isolated adult satellite cells [21, 22] with a diverse chemical library to identify compounds that promote expansion. We chose this metric for our screen as this phenotypic outcome is broadly applicable to age-, disease-, or injury-induced muscle degeneration.

Satellite cell proliferation can be modulated by multiple signaling pathways, including FGF [17], TGF-β [54], p38 [55, 56], JAK/STAT [30, 57], Wnt signaling [58, 59] and Prostaglandin E2 [16]. In this study, we identified several compounds annotated as inhibitors of different receptor tyrosine kinases including PDGFR, FLT3, RET, and CSFR-1. CEP-701, the compound in this class that we investigated most thoroughly, was capable of expanding mouse and human satellite cells in the nanomolar range. Interestingly, CEP-701 is best known as a FLT3 kinase inhibitor and effectively suppresses the proliferation of myeloid leukemia cells [39, 40]. We found that *Flt3* was not expressed appreciably in satellite cells and genetic deletion of *Flt3* did not improve satellite cell proliferation or impact the pro-expansion effects on these cells of CEP-701. As such, we explored other RTKs expressed by satellite cells and identified the proto-oncogene RET as a strong candidate target for CEP-701 inhibition. RET serves as a receptor for members of the GDNF family of ligands (GFLs) [60], made up of GDNF, neurturin (Nrtn), persephin (Pspn), and artemin (Artn) [61]. Our data implicate the upregulation of RET protein levels as a distinguishing feature following satellite cell activation and demonstrate that CEP-701 inhibits RET signaling in satellite cells. Furthermore, we show that systemic delivery of CEP-701 via subcutaneous injection augments the number of satellite cells present in damaged skeletal muscle and accelerates the kinetics of muscle regeneration. Similarly, the expansion of satellite cells ex vivo in the presence of CEP-701 enhanced their engraftment potential following transplantation. Taken together, these data strongly support the conclusion that transient treatment with CEP-701 promotes skeletal muscle regeneration at least in part through inhibition of RET signaling in satellite cells while they expand.

RET/GFL signaling has reported roles in muscle maintenance, development, and adaptation to exercise. GDNF is constitutively secreted by skeletal muscle fibers and is involved in muscle regeneration and maintenance of the neuromuscular junction [62–64]. Furthermore, GDNF is upregulated following increased physical activity in rats [65] and in disease models of muscular dystrophy and polymyocitis [62]. However, mechanisms by which RET/GFL signaling directly regulates satellite cell activation or self-renewal have not been well studied. We show that 10% of Pax7+ cells express RET at the time of isolation and that it is dramatically upregulated after isolation, during the period in which we carry out compound treatment, and when satellite cell activation and the first cell divisions are occurring. Together, our results suggest that GDNF and RET could be playing roles in the quiescent satellite cell itself, and/or following activation, in the ability of satellite cells to self-renew and return to quiescence, and that transient inhibition of RET phosphorylation by CEP-701 treatment results in satellite cell pool expansion. This finding is important as it suggests that inhibition of a specific subfamily of RTKs, in this case, type III RTKs, results in the expansion of satellite cells. Inhibition of RET signaling provides an upstream target capable of modulating simultaneously two of the major pathways involved in satellite cell expansion—namely the p38 and JAK/STAT pathways. This also speaks to the complexity of the actions of RTKs in the myogenic lineage as other RTK ligands have been implicated in the transition to the activated state (HGF [66]), enhanced expansion of activated satellite cells and the inhibition of differentiation (FGF [67]) and muscle fiber hypertrophy (IGF1 [68]).

A recent report by Li et al. using genetic perturbation of both RET and GAS1, an endogenous RET inhibitor, [69] showed that long-term RET inhibition (accomplished by genetic deletion of RET or GAS1 overexpression) led to decreased numbers of satellite cells in vivo and smaller regenerating muscle fibers. At first glance, these results seem to be in conflict with ours. We believe these results are best reconciled by exploring the timing and duration of RET inhibition. As satellite cells become activated, they go through a period of cell division and expansion giving rise to committed myoblasts which continue along the myogenic program, eventually fusing with myofibers. In parallel, satellite cells are capable of self-renewal and eventually return to quiescence within their niches. We treated freshly isolated satellite cells with CEP-701 for 96 h or less. This acute inhibition of RET limited its effects on quiescence and self-renewal, leading to more robust activation and expansion in the short term. However, prolonged inhibition of RET (and by extension, the prolonged inhibition of quiescence and self-renewal) would be expected to cause greater numbers of activated satellite cells initially, before eventually depleting the satellite cell pool, as was seen by Li et al. following genetic inhibition of RET signaling for 1 month. This exact pattern of expansion followed by exhaustion was also shown for HIF2A where transient genetic or pharmacological inhibition leads to increased satellite cell numbers and improved regeneration, but sustained inhibition leads to satellite cell depletion and impaired regeneration [70].

Moyle et al. [42] also studied the effects of RET inhibition on myogenic cells. These authors demonstrated that knockdown of RET using siRNAs did not cause myoblasts to proliferate, but it did enhance their fusion, as did in vitro treatment with sunitinib. Additionally, in contrast to the inhibitory role RET plays in satellite cell expansion, constitutive activation of RET signaling, or treatment with GDNF, increased proliferation of myoblasts [42]. These findings are also in line with work carried out in the immortalized myoblast line C2C12 [71]. In that work, the authors showed that GAS1 overexpression leads to enhanced C2C12 fusion, again implicating these pathways in the generation of differentiated myotubes. These studies are consistent with our observations showing that while CEP-701 and sunitinib can expand freshly isolated satellite cells in vitro (Fig. 2), they enhanced myoblast fusion without changing cell numbers during differentiation (Fig. 4).

Consideration of these results, together with the data reported in our study, strongly suggests that the timing and duration of RET inhibition will be of critical importance for the application of these findings in a therapeutic context. While an acute pharmacologic reduction of RET signaling post-injury might accelerate the regenerative process, chronic ablation of RET signaling could lead to precocious differentiation of transit-amplifying progenitors and subsequent depletion of the endogenous satellite cell pool via the premature exit from the quiescent state and/or the failure to self-renew [69]. Ultimately, our results, along with emerging data in the field, identify an unexpected beneficial role for transient pharmacological inhibition of RET in satellite cell activation and further underscore the dynamic roles GDNF/RET play during the various stages of skeletal muscle regeneration.

## Conclusions

Our data uncovered a role for CEP-701-mediated RET inhibition in the context of satellite cell expansion both in vitro and in vivo and identified several RTK inhibitors (CEP-701, sunitinib, bosutinib, and SU11652) that affect satellite cell expansion. Our experiments provide compelling evidence that pharmacological inhibition of this target in vivo may present a viable therapeutic approach to stimulate the kinetics of muscle regeneration. Furthermore, our approach outlines a platform to identify therapeutically relevant compounds that regulate satellite cell expansion in vitro, opening the possibility of identifying and developing additional satellite cell-directed therapies to improve muscle function after damage or disease.

## Supplementary information

**Additional file 1: Supplementary Figure 1.** (A) Freshly isolated satellite cells (SC) or committed myoblasts (Myo) were seeded at 2000 cells/well for 24 hours, fixed and stained for Pax7 and MyoD expression. (B) Quantification of percent of cells expressing Pax7, MyoD or both at levels above threshold. n=2-3 biological replicates, minimum 1995 and maximum 18,224 cells analyzed per replicate. (C) SC and Myo were seeded as above and stained for the proliferation marker Ki67. (D) Quantification of percent of cells expressing nuclear Ki67 above threshold. n=2-3 biological replicates, minimum 868 and maximum 9071 cells analyzed per replicate. (E) SC and Myo were seeded as above and actin was stained with phalloidin conjugated to Alexa-488. (F) Quantification of mean cell area of freshly sorted satellite cells and myoblasts. n=2-3 biological replicates, minimum 821 and maximum 9117 cells analyzed per replicate. Scale bars indicate 100μm. n.s.: not significant, **p<0.01, ***p<0.001 by an unpaired two-tailed t test assuming unequal variance.

**Additional file 2: Supplementary Figure 2.** (A) Experimental schematic outlining the in vitro treatment of human satellite cells with CEP-701. (B)-(H) Expansion of human satellite cells isolated from individual donors and cultured in vitro in the presence or absence of CEP-701. CEP-701 significantly increases proliferation of cells from Donors 1, 2, 3 and 7, while cells from Donors 4 and 6 show a trend towards increased proliferation. *p < 0.05, **p < 0.01, ***p < 0.001 by 1-way ANOVA followed by unpaired two-tailed t test assuming unequal variance with Bonferroni correction for multiple comparisons.

**Additional file 3: Supplementary Figure 3.** (A) eMHC stain (green) is specific to regenerating myofibers in injured muscle. Tibialis anterior muscle was stained for laminin and eMHC after cardiotoxin injury (Injured) or no treatment control (Contralateral) and regenenerating eMHC^+^ fibers were identified (inset). Scale bar indicates 500μm. (B) Frequency distribution of cross-sectional areas of individual eMHC^+^ regenerating myofibers in mice treated with vehicle or 10mg/kg CEP-701. ***p<0.001 by an unpaired two-tailed t test assuming unequal variance. (C) Quantification of the fold change in fibro-adipogenic precursor cells (FAPs) in regenerating muscle following treatment with CEP-701. TA muscle was damaged with CTX and animals were treated subcutaneously, twice a day with vehicle or 10mg/kg CEP-701. SCA1^+^ FAPs were isolated by FACS and quantified as a percentage of the total calcein AM^+^/propidium iodide^-^ live cells. Error bars indicate SEM from 7 independent experiments. **p < 0.01 by an unpaired two-tailed t test assuming unequal variance. (D) Quantification of the fold change in blood-lineage/immune cells in regenerating muscle following treatment with CEP-701. TA muscle was damaged with CTX and animals were treated subcutaneously, twice a day with vehicle or 10mg/kg CEP-701. CD11b^+^, TER119^+^ and CD45^+^ blood lineage cells were isolated in aggregate by FACS and quantified as a percentage of the total calcein AM^+^/propidium iodide^-^ live cells. Error bars indicate SEM from 7 independent experiments. **p < 0.01 by an unpaired two-tailed t test assuming unequal variance.

**Additional file 4: Supplementary Figure 4.** (A) CEP-701 and sunitinib inhibit the growth of the acute monocytic leukemia cell line THP-1. Cells were grown in the presence of the indicated concentrations of compound for 7 days and proliferation was assessed by MTT assay. (B) CEP-701 and sunitinib inhibit the growth of the neuroblastoma cell line Neuro-2a. Cells were grown in the presence of the indicated concentrations of compound for 7 days and proliferation was assessed by high content imaging.

**Additional file 5: Supplementary Figure 5.** (A) Relative fold change in the mean number of satellite cells/well of wild type or *FLT3* knock out (*FLT3*^*-/-*^) mice following 6 days in culture in the presence or absence of 50nM CEP-701 or DMSO control. * p value <0.05 by 1 way ANOVA followed by unpaired t-test with Bonferroni correction. (B) Percentage of satellite cells expressing RET protein post-fiber isolation (0hrs) on single muscle fibers isolated from EDL muscle of adolescent (3 week) Tg:Pax7^nGFP^ mice. Data represent mean ± SD from 3 independent experiments. Statistical significance was evaluated by an unpaired two-tailed t test assuming unequal variance relative to adult (Pax7^+^/RET^+^) 0hrs. (***p < 0.001). (C) Quantification of mean GFRα1 intensity per well in CXCR4^+^/ITGβ1^+^ satellite cells following 24, 48 and 72hrs of culture. Error bars indicate SD from 3 independent experiments. Statistical significance was evaluated by an unpaired two-tailed t test assuming unequal variance, (**p < 0.01).

**Additional file 6: Supplementary table.** Primary Screen Data.

## Data Availability

Datasets generated and analyzed in this study are included here or available from the authors upon request.

## Abbreviations

bFGF: Basic fibroblast growth factor
CEP-701: Lestaurtinib
CTX: Cardiotoxin
DMSO: Dimethylsulfoxide
EDL: Extensor digitorum longus
EGFP: Enhanced green fluorescent protein
eMHC: Embryonic myosin heavy chain
FAPs: Fibroadipogenic progenitors
FBS: Fetal bovine serum
FLT3: FMS-related receptor tyrosine kinase 3
GAS1: Growth arrest-specific protein 1
GDNF: Glial cell-derived neurotrophic factor
GFP: Green fluorescent protein
GFRA1: GDNF family receptor alpha 1
GPCR: G protein-coupled receptor
Ki67: Marker of proliferation Ki-67
MyHC: Myosin heavy chain
MyoD: Myoblast determination protein 1
PAX7: Paired box 7
RET: Rearranged during transfection proto-oncogene
RTK: Receptor tyrosine kinase
STITCH: Search tool for interactions of chemicals
TA: Tibialis anterior
TGF-β: Transforming growth factor beta
