# Supplementary figures and images for: Pro-myogenic small molecules revealed by a chemical screen on primary muscle stem cells
Source: Skelet Muscle. 2020 Oct 9;10:28. doi: 10.1186/s13395-020-00248-z (PMC7547525; doi:10.1186/s13395-020-00248-z)

Supplementary Figure 1.

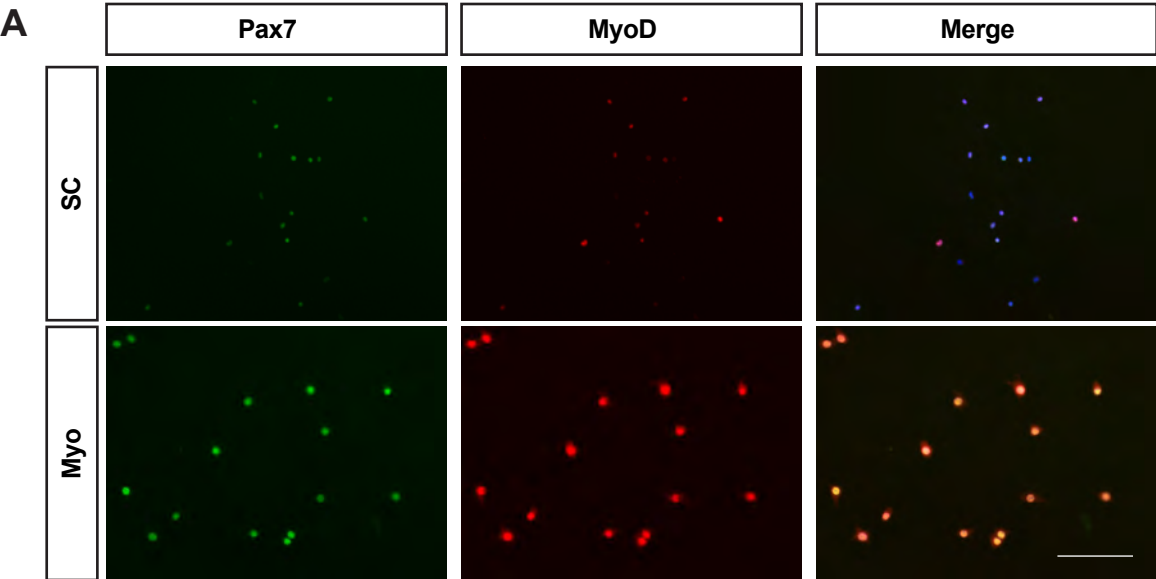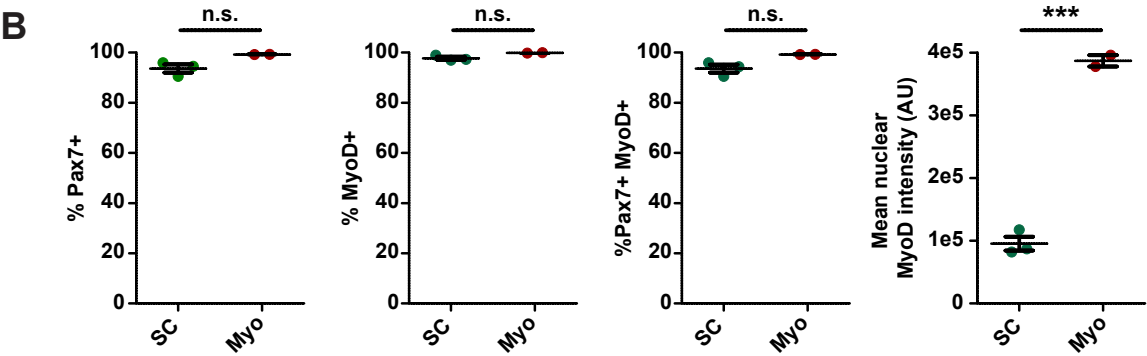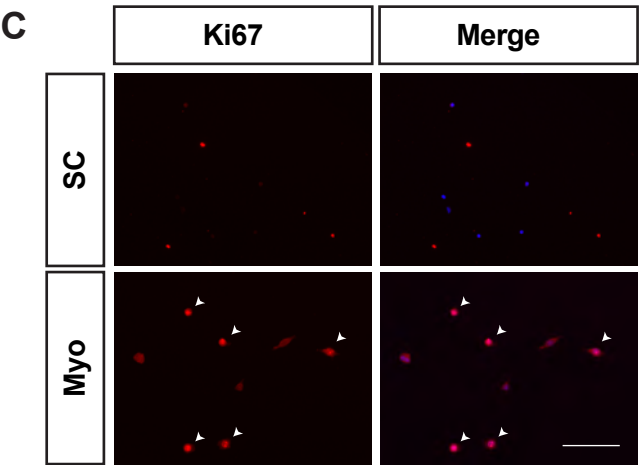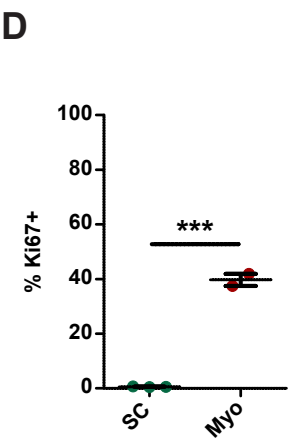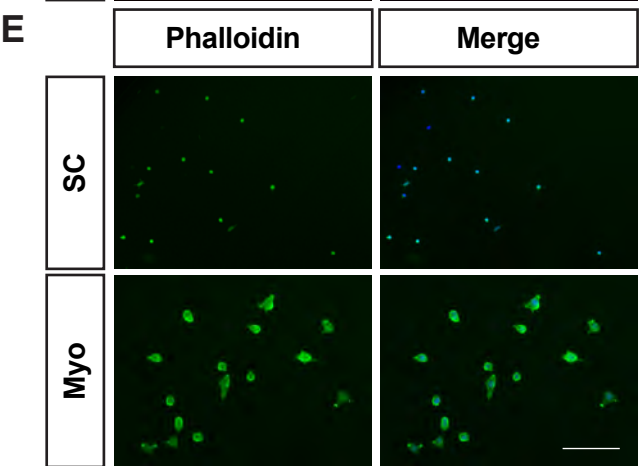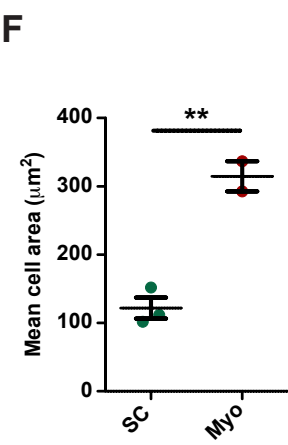

Supplement: Supplementary file 1 — Additional file 1: Supplementary Figure 1. (A) Freshly isolated satellite cells (SC) or committed myoblasts (Myo) were seeded at 2000 cells/well for 24 hours, fixed and stained for Pax7 and MyoD expression. (B) Quantification of percent of cells expressing Pax7, MyoD or both at levels above threshold. n=2-3 biological replicates, minimum 1995 and maximum 18,224 cells analyzed per replicate. (C) SC and Myo were seeded as above and stained for the proliferation marker Ki67. (D) Quantification of percent of cells expressing nuclear Ki67 above threshold. n=2-3 biological replicates, minimum 868 and maximum 9071 cells analyzed per replicate. (E) SC and Myo were seeded as above and actin was stained with phalloidin conjugated to Alexa-488. (F) Quantification of mean cell area of freshly sorted satellite cells and myoblasts. n=2-3 biological replicates, minimum 821 and maximum 9117 cells analyzed per replicate. Scale bars indicate 100μm. n.s.: not significant, **p<0.01, ***p<0.001 by an unpaired two-tailed t test assuming unequal variance. [file 13395_2020_248_MOESM1_ESM.pdf]

Supplementary Figure 2.

A

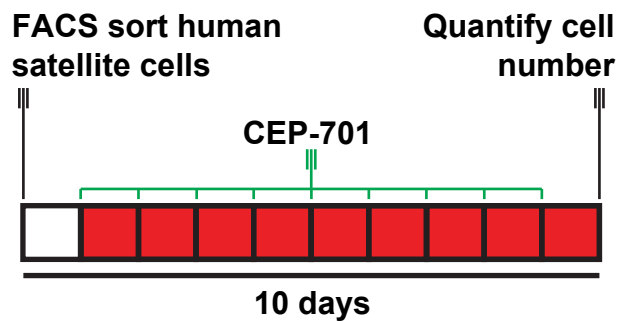

B

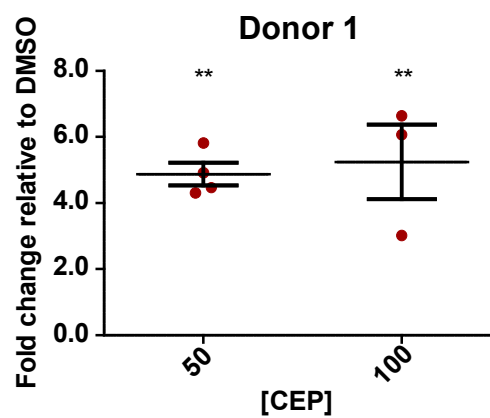

C

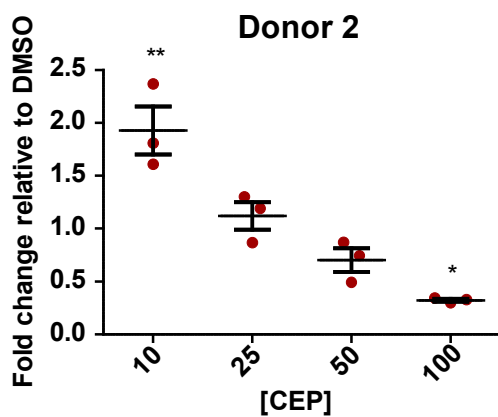

D

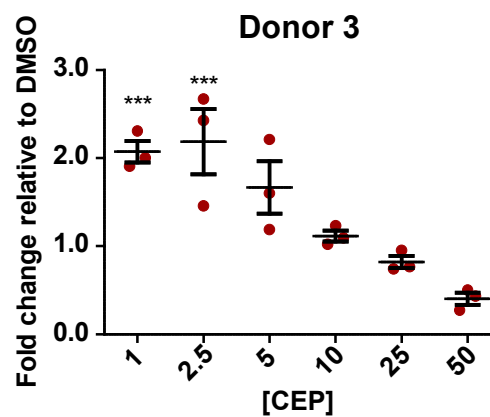

E

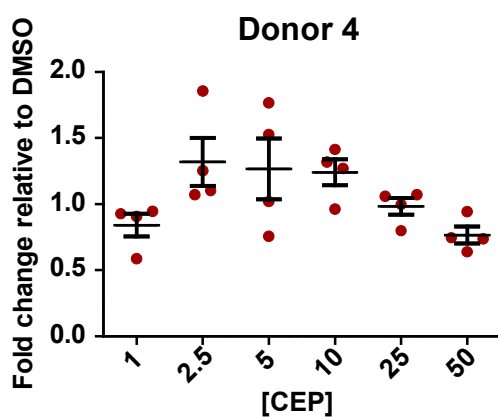

F

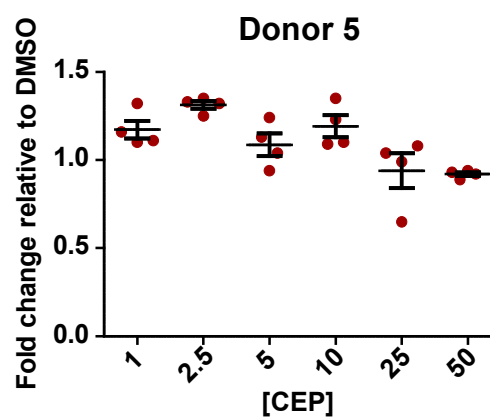

G

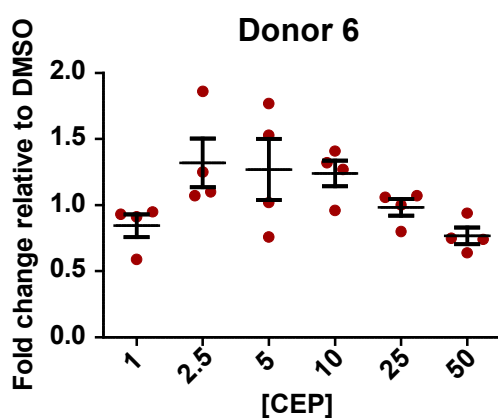

H

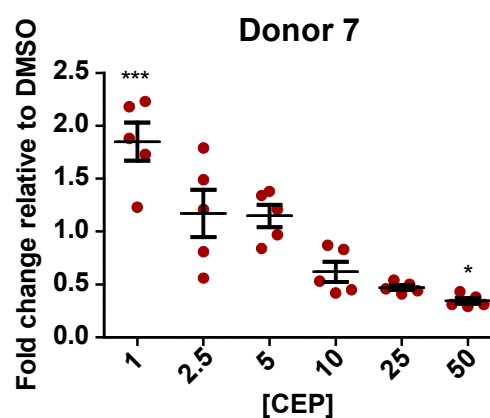

Supplement: Supplementary file 2 — Additional file 2: Supplementary Figure 2. (A) Experimental schematic outlining the in vitro treatment of human satellite cells with CEP-701. (B)-(H) Expansion of human satellite cells isolated from individual donors and cultured in vitro in the presence or absence of CEP-701. CEP-701 significantly increases proliferation of cells from Donors 1, 2, 3 and 7, while cells from Donors 4 and 6 show a trend towards increased proliferation. *p < 0.05, **p < 0.01, ***p < 0.001 by 1-way ANOVA followed by unpaired two-tailed t test assuming unequal variance with Bonferroni correction for multiple comparisons. [file 13395_2020_248_MOESM2_ESM.pdf]

Supplementary Figure 3.

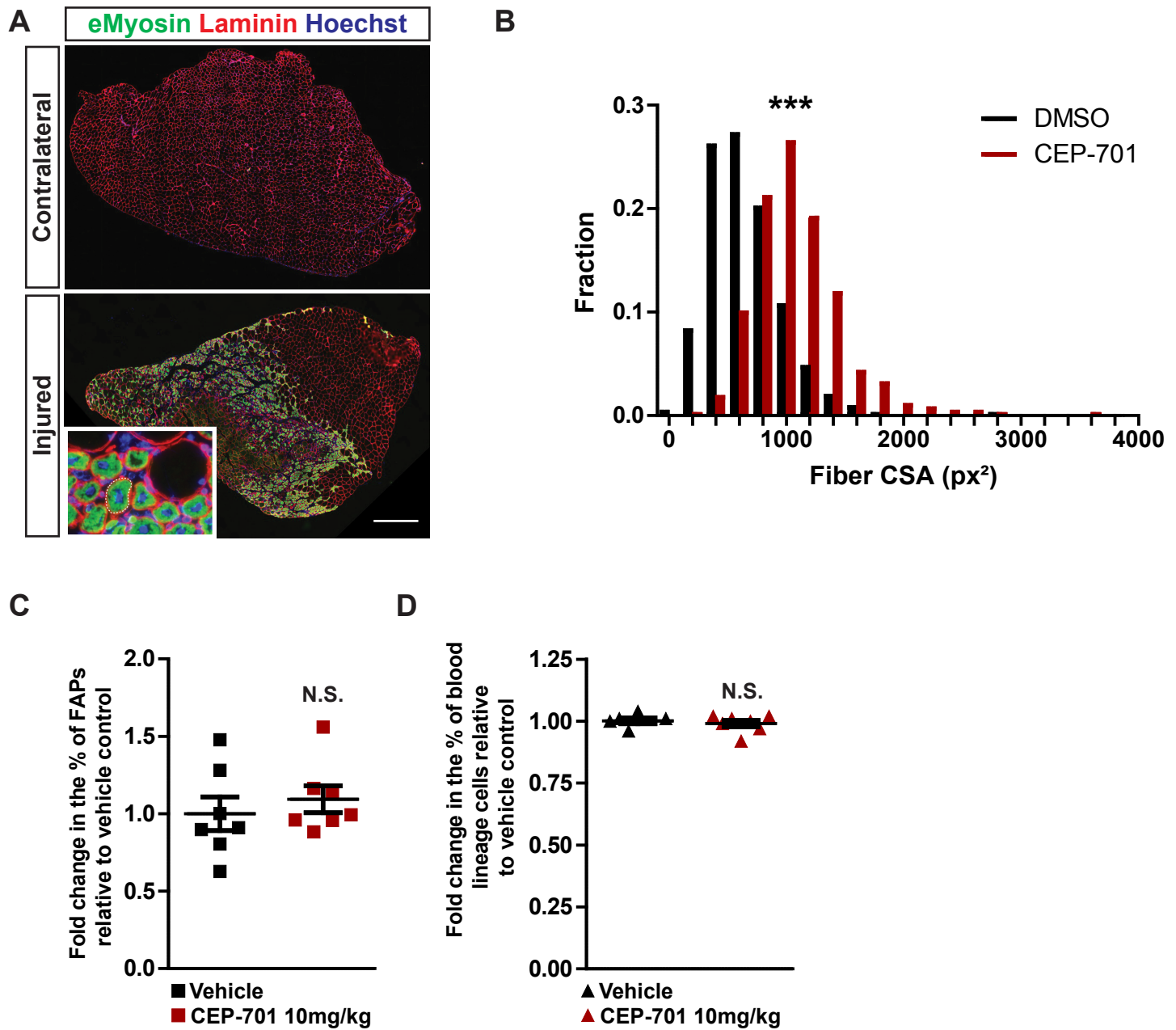

Supplement: Supplementary file 3 — Additional file 3: Supplementary Figure 3. (A) eMHC stain (green) is specific to regenerating myofibers in injured muscle. Tibialis anterior muscle was stained for laminin and eMHC after cardiotoxin injury (Injured) or no treatment control (Contralateral) and regenenerating eMHC+ fibers were identified (inset). Scale bar indicates 500μm. (B) Frequency distribution of cross-sectional areas of individual eMHC+ regenerating myofibers in mice treated with vehicle or 10mg/kg CEP-701. ***p<0.001 by an unpaired two-tailed t test assuming unequal variance. (C) Quantification of the fold change in fibro-adipogenic precursor cells (FAPs) in regenerating muscle following treatment with CEP-701. TA muscle was damaged with CTX and animals were treated subcutaneously, twice a day with vehicle or 10mg/kg CEP-701. SCA1+ FAPs were isolated by FACS and quantified as a percentage of the total calcein AM+/propidium iodide- live cells. Error bars indicate SEM from 7 independent experiments. **p < 0.01 by an unpaired two-tailed t test assuming unequal variance. (D) Quantification of the fold change in blood-lineage/immune cells in regenerating muscle following treatment with CEP-701. TA muscle was damaged with CTX and animals were treated subcutaneously, twice a day with vehicle or 10mg/kg CEP-701. CD11b+, TER119+ and CD45+ blood lineage cells were isolated in aggregate by FACS and quantified as a percentage of the total calcein AM+/propidium iodide- live cells. Error bars indicate SEM from 7 independent experiments. **p < 0.01 by an unpaired two-tailed t test assuming unequal variance. [file 13395_2020_248_MOESM3_ESM.pdf]

Supplementary Figure 4.

A

THP-1

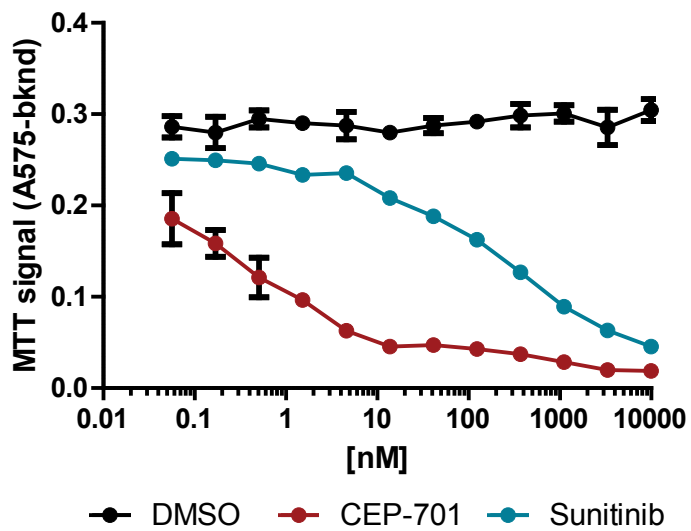

B

N2a

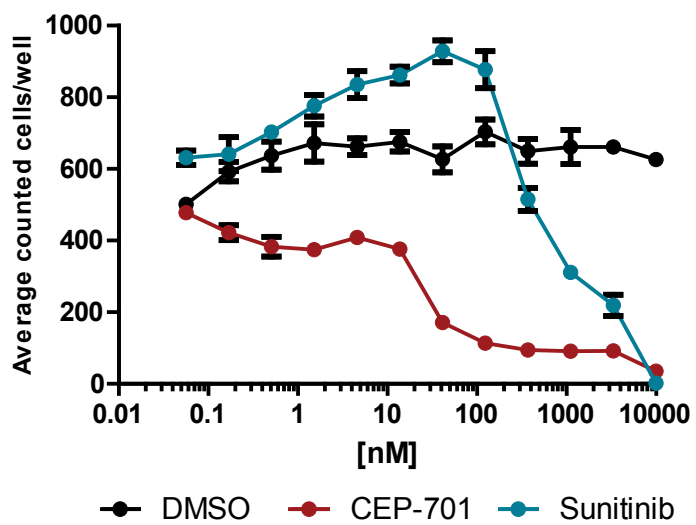

Supplement: Supplementary file 4 — Additional file 4: Supplementary Figure 4. (A) CEP-701 and sunitinib inhibit the growth of the acute monocytic leukemia cell line THP-1. Cells were grown in the presence of the indicated concentrations of compound for 7 days and proliferation was assessed by MTT assay. (B) CEP-701 and sunitinib inhibit the growth of the neuroblastoma cell line Neuro-2a. Cells were grown in the presence of the indicated concentrations of compound for 7 days and proliferation was assessed by high content imaging. [file 13395_2020_248_MOESM4_ESM.pdf]

Supplementary Figure 5.

A

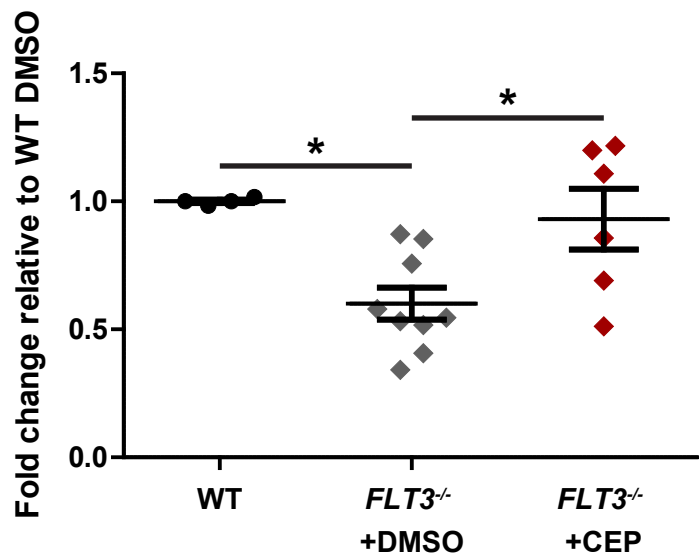

B

Time 0hrs - 3 week old EDL fiber

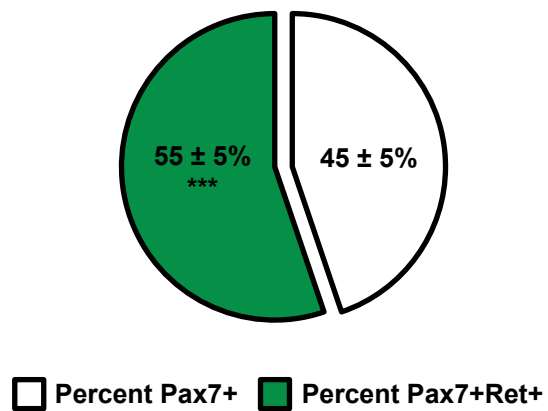

C

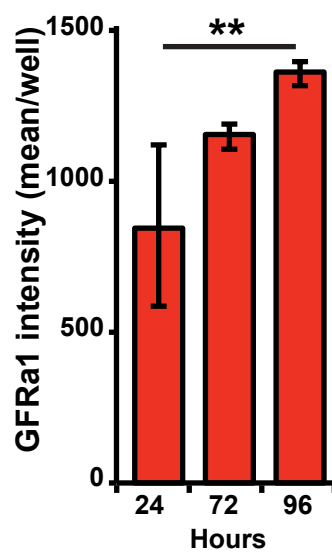

Supplement: Supplementary file 5 — Additional file 5: Supplementary Figure 5. (A) Relative fold change in the mean number of satellite cells/well of wild type or FLT3 knock out (FLT3-/-) mice following 6 days in culture in the presence or absence of 50nM CEP-701 or DMSO control. * p value <0.05 by 1 way ANOVA followed by unpaired t-test with Bonferroni correction. (B) Percentage of satellite cells expressing RET protein post-fiber isolation (0hrs) on single muscle fibers isolated from EDL muscle of adolescent (3 week) Tg:Pax7nGFP mice. Data represent mean ± SD from 3 independent experiments. Statistical significance was evaluated by an unpaired two-tailed t test assuming unequal variance relative to adult (Pax7+/RET+) 0hrs. (***p < 0.001). (C) Quantification of mean GFRα1 intensity per well in CXCR4+/ITGβ1+ satellite cells following 24, 48 and 72hrs of culture. Error bars indicate SD from 3 independent experiments. Statistical significance was evaluated by an unpaired two-tailed t test assuming unequal variance, (**p < 0.01). [file 13395_2020_248_MOESM5_ESM.pdf]
